# Tooth-Brushes

**Published:** 1869-11

**Authors:** 


TOOTH-BRUSHES.—There has lately been introduced into the market a porous form of vulcanized india rubber, called india-rubber sponge. It is proposed to substitute this material for bristles in the manufacture of tooth-brushes. A piece of india-rubber sponge is fixed to a handle of bone or ivory, and ridges are formed on the surface of the spongy material. Other brushes are made in a similar manner by fixing spongy vulcanized india-rubber to a rigid back or handle; or, in some cases, as for horse-brushes, a rigid back only is required. In some cases, the spongy india-rubber is checkered or cross-grooved.

